# Estimation of timing of infection from longitudinal SARS-CoV-2 viral load data: mathematical modelling study

**DOI:** 10.1186/s12879-022-07646-2

**Published:** 2022-07-28

**Authors:** Keisuke Ejima, Kwang Su Kim, Ana I. Bento, Shoya Iwanami, Yasuhisa Fujita, Kazuyuki Aihara, Kenji Shibuya, Shingo Iwami

**Affiliations:** 1 Department of Epidemiology and Biostatistics, Indiana University School of Public Health-Bloomington, Bloomington, IN USA; 2 The Tokyo Foundation for Policy Research, Tokyo, Japan; 3 Interdisciplinary Biology Laboratory, Division of Natural Science, Graduate School of Science, Nagoya University, Nagoya, Japan; 4 Department of Science system simulation, Pukyong National University, Busan, South Korea; 5 International Research Center for Neurointelligence, The University of Tokyo, Tokyo, Japan; 6 Institute of Mathematics for Industry, Kyushu University, Fukuoka, Japan; 7 Institute for the Advanced Study of Human Biology (ASHBi), Kyoto University, Kyoto, Japan; 8 NEXT-Ganken Program, Japanese Foundation for Cancer Research (JFCR), Tokyo, Japan; 9 Interdisciplinary Theoretical and Mathematical Sciences Program (iTHEMS), RIKEN, Saitama, Japan; 10 Science Groove Inc., Fukuoka, Japan

**Keywords:** SARS-CoV-2, COVID-19, Mathematical model, Viral load, Contact trace

## Abstract

**Background:**

Multiple waves of the COVID-19 epidemic have hit most countries by the end of 2021. Most of those waves are caused by emergence and importation of new variants. To prevent importation of new variants, combination of border control and contact tracing is essential. However, the timing of infection inferred by interview is influenced by recall bias and hinders the contact tracing process.

**Methods:**

We propose a novel approach to infer the timing of infection, by employing a within-host model to capture viral load dynamics after the onset of symptoms. We applied this approach to ascertain secondary transmission which can trigger outbreaks. As a demonstration, the 12 initial reported cases in Singapore, which were considered as imported because of their recent travel history to Wuhan, were analyzed to assess whether they are truly imported.

**Results:**

Our approach suggested that 6 cases were infected prior to the arrival in Singapore, whereas other 6 cases might have been secondary local infection. Three among the 6 potential secondary transmission cases revealed that they had contact history to previously confirmed cases.

**Conclusions:**

Contact trace combined with our approach using viral load data could be the key to mitigate the risk of importation of new variants by identifying cases as early as possible and inferring the timing of infection with high accuracy.

**Supplementary Information:**

The online version contains supplementary material available at 10.1186/s12879-022-07646-2.

## Background

Most countries have experienced multiple waves of COVID-19 epidemic by the end of 2021. Those different waves are due to emergence and importation of new variants of SARS-CoV-2. Further, given that waning immunity is inevitable even after infection or (multiple doses of) vaccination [1–5], more waves of the epidemic are anticipated to happen in the next few years [6].

There are three major control knobs to prevent a resurgence: border control; test, trace contacts and isolate; and social distancing measures [7]. To avoid future outbreaks, governments may need to implement various border control programs: quarantine of suspicious cases and isolation of confirmed cases, and travel restriction to and from countries with ongoing outbreaks. Border control, which is generally based on symptoms, cannot capture all cases given approximately a 5-day incubation period and non-negligible number of asymptomatic cases of COVID-19 (i.e., cases can pass the control program if they are not symptomatic) [8–16]. Matteo and colleagues examined that travel border control only delayed epidemic progression by 3–5 days within China, suggesting border control solely cannot prevent outbreaks [17]. Therefore, “test, contact trace and isolate” measure plays an essential role in controlling local transmission and reducing the risk of subsequent outbreaks.

Contact trace starts once cases are confirmed. Confirmed cases are followed by further investigation–through interviews, contact tracing, and genomic analysis in some cases–to assess when and where they were infected [18, 19]. This tells us a lot about the transmission mode and benefits contacts. First, contact trace benefits cases with better clinical outcomes. Bi and colleagues reported that the cases identified by contact trace were identified and treated earlier than cases identified by symptom-based survey in Shenzhen, China [20]. Second, it is used to identify highly transmissible situation. For example, Nishiura and colleague found SARS-CoV-2 is highly transmissible in closed environment through contact trace data [21]. The third, and what we will focus on in this study, is that contact trace is used to ascertain whether the cases are locally transmitted or imported.

Epidemics are triggered by importation of new cases. Indeed, Kathy and colleagues reported that the first wave was initiated by imported cases from Hubei province to mega cities such as Beijing, and the further local transmissions created the first wave [22]. Interestingly, new cases reported afterwards were imported from overseas, suggested potential risk of further outbreak in China. Thus many studies separately reported imported cases and cases of local transmission [22, 23]. Ascertaining whether new COVID-19 cases are imported or due to local secondary transmission is essential for a government to develop public health strategies. If they are the latter and the number of local transmissions is substantial, outbreaks are inevitable, and the goal of intervention programs should be shifted from containment to mitigation.

Traditionally, the ascertainment of local transmission requires interview-based assessments, which would be time-consuming and potentially biased because the timing of infection is not directly observable in many cases (extremely hard for SARS-CoV-2 because it is an airborne disease [24–26]) except some special cases with specific known infection route such as HIV [27, 28]. As such, most of the studies inferring the timing of infection for COVID-19 were based on active surveillance or contact trace [8–11]. However, if cases cannot report correct day(s) of exposure, the investigation could be biased and the source of infection will not be identified. Especially for COVID-19, the bias could be worsen given relatively long incubation period of COVID-19 compared with influenza [17]. For example, if ones travel frequently during the long incubation period, they may not remember when and where they were exposed. Indeed, Lauer et al. and Bi et al. demonstrated large uncertainly on the estimation of the timing of infection for COVID-19 in China [8, 20]. Even accounting for uncertainty for the timing of infection with exposure information using Bayesian approach when estimating incubation period [9, 29], bias is not perfectly removed. Further, given that there is substantial portion of pre-symptomatic and asymptomatic infection [12–16], specification of the exposure events is challenging even with stringent contact tracing protocol.

Mathematical models have been used to study infectious diseases including COVID-19 at any scale from the population level to the within-host level [30]. Especially, between-hosts models are widely adopted to describe the transmission process (between hosts) of an infection, study the epidemiology of infectious diseases, and assess the effectiveness of different intervention measures at the population level [31–34]. These models found ample adoption by public health officials and governments in a number of ways that range from situational awareness to intervention planning and projections [35] as well as for filling data gaps, estimating key epidemiological parameters (such as the reproduction number), and explaining the mechanisms behind the observed patterns [20]. On the other hand, the model with another scale, within-host models have been developed to describe the process of viral replication and removal as well as immune response within a single person [36]. Overall, these models capture both the viral dynamics within a host while it is infected as well as changes in immune level after infection (e.g., waning of immunity, boosting). Furthermore, these models can be used to describe and test personalized interventions through in-silico experiments, providing insights, for instance, on the development of treatments and treatment regimens, timing of booster vaccination, and clinical trial design [37, 38]. Within-host models allow considering the intrinsic biological difference between patients, which may alter the epidemiology and transmission patterns of an infectious disease outbreak (as observed for SARS-CoV-2 infection [16, 39–41]).

In this study, we propose to use the viral dynamics model to differentiate secondary local transmission from imported cases, which complements contact trace and reduces its inherent bias. For the purpose of illustration, we analyzed cases in the early phase of the first wave in Singapore.

## Methods

**Fig. 1:**
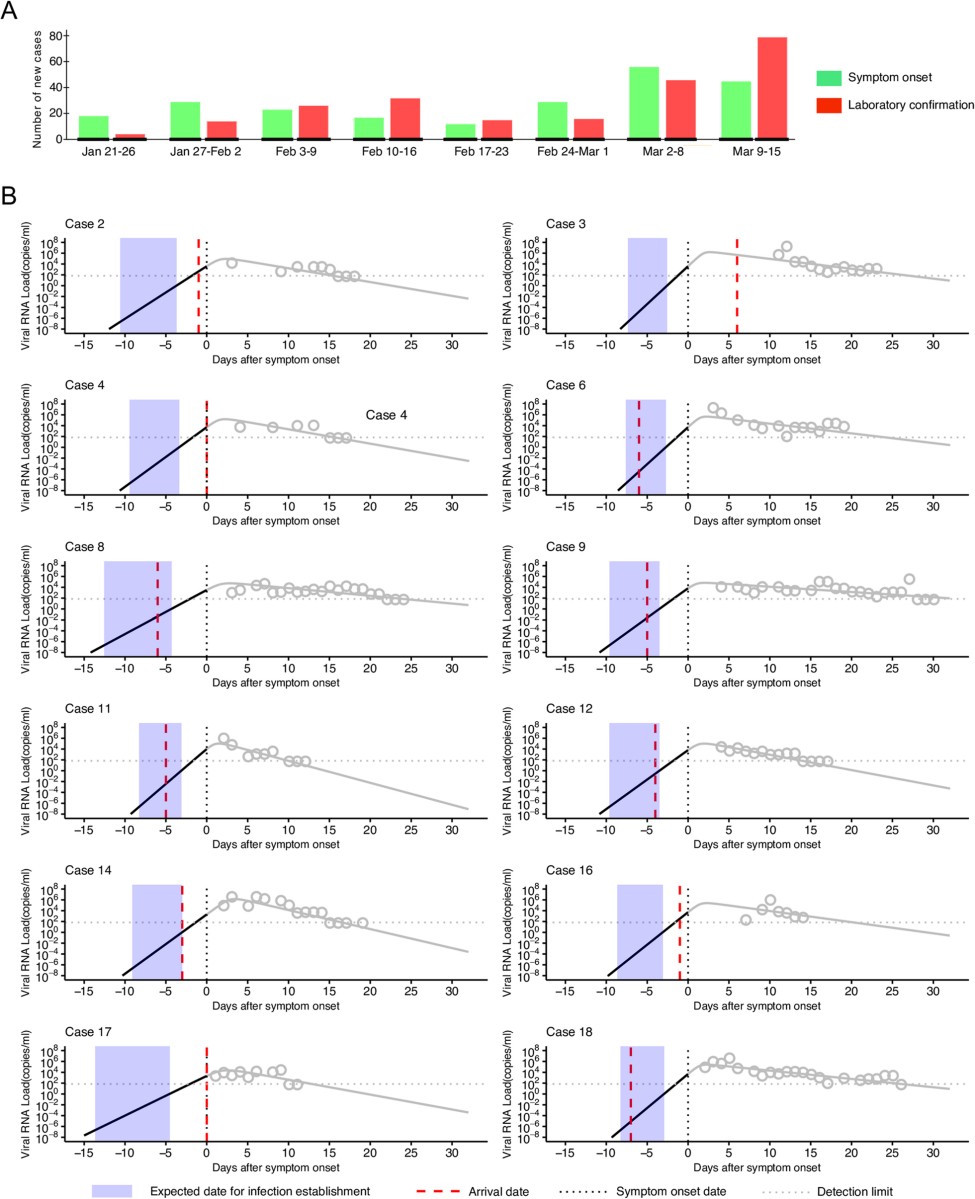
Epidemic curve of COVID-19 and clinical course of patients in Singapore. **A** Epidemic curves of COVID-19 as of March 10, 2020 in Singapore are shown. The green and red solid bars correspond to the newly reported cases by date of symptom onset and by date of laboratory confirmation, respectively. **B** Each panel presents timeline of infection for each case. Expected SARS-CoV-2 viral dynamics and observed viral load for the first 13 cases are depicted by grey (or black) solid lines and grey open circles, respectively. The timing of arrival to Singapore (red dashed lines), the timing of symptom onset (black dashed lines), the estimated timing of infection establishment (blue shaded areas), and the detection limit of viral load (grey dashed lines) are also shown

In Singapore, the first case was identified on 23rd January 2020 (Fig. 1A). The first 18 cases had travel history to Wuhan, China, thus were considered to be imported cases[42]. Two days after the 18th case was confirmed (3rd February 2020), a new case was identified which had no history of traveling to China. To investigate the possibility of some of the original 18 being evidence of ongoing local transmission, we leveraged viral load data collected[42] for multiple time points after the onset of symptoms using a within-host viral dynamics model for SARS-CoV-2. This enables us to infer time of infection (i.e., before or after arrival to Singapore).

### Viral load data

We extracted data from two published papers [42, 43] (we have not collected original data in this study). Nasopharyngeal swabs were collected from the 18 cases reported for up to 30 days from the onset of symptoms. Viral loads were measured by RT-PCR[42]. We excluded 5 cases who received antivirals (i.e., lopinavir, ritonavir) because the antiviral effect could not be quantified with the limited data, and a case whose viral load was detected only twice (i.e., not enough to hindcast the viral load trajectory). In total, we analyzed the first 12 cases. In order to find “infection establishment boundary” (see “Viral load boundary for infection establishment” section) and derive robust parameter estimation, we obtained an additional dataset of viral loads measured in nasal swab collected from the 8 cases reported in Zhuhai, China[43]. Three of these cases were confirmed as secondary infections and their days of infection were reported, thus we used the information to compute the viral load threshold for the infection establishment.

Cycle threshold (Ct) values reported in Zou et al*.* [43] and Young et al. [42], which are the cycle numbers when the fluorescent signal crosses the threshold, were converted to viral RNA copies (copies/mL); these quantities were shown to be inversely proportional to each other from a previous SARS-CoV study [44]. The values under the detection limit were assumed to be at the detection limit for the purposes of fitting the model (see later for detail). We used the program datathief III (version 1.5, Bas Tummers, www.datathief.org) to extract the data from images in those publications. Waiver of informed consent was granted by public health authorities or written informed consent was obtained from study participants as described in the original studies.

### Viral load modelling to estimate the day of infection establishment

Based on a standard viral dynamics model, to describe SARS-CoV-2 dissemination among target cells, we used the following simple mathematical model previously proposed [45–47]:

1 $$\frac{df(t)}{dt}=-\beta f\left(t\right)V\left(t\right), \frac{dV\left(t\right)}{dt}=\gamma f\left(t\right)V\left(t\right)-\delta V\left(t\right),$$

where $f(t)$ and $V(t)$ are the ratio of uninfected target cells and the amount of virus, respectively. The parameters $\beta$, $\gamma$, and $\delta$ represent the rate constant for virus infection, the maximum rate constant for viral replication, and the death rate of infected cells, respectively. All viral load data including Singapore and Zhuhai patients were simultaneously fitted using a nonlinear mixed-effect modelling approach, which uses the whole samples to estimate population parameters while accounting for inter-patient variation.

Further, sampled parameter sets were used to predict the estimated day of SARS-CoV-2 infection establishment, that is, the start of the exponential growth phase of viral loads [47]. The infection establishment time, ${T}_{inf}$, was estimated by hindcasting the mathematical model, when the viral load reaches a boundary. The viral load boundary for infection establishment was computed using the three secondary infection cases reported in Zhuhai, whose initial days of exposure to the primary cases are known [43]. We assumed that the initial day of exposure is equal to the day of infection establishment. Once the day of infection establishment is obtained for each case, it was compared against the date of arrival in Singapore. If the estimated day of infection is before the arrival in Singapore, it suggests that the infection was established outside Singapore, otherwise, the case is the result of secondary local transmission within Singapore.

### Viral load boundary for infection establishment

**Fig. 2:**
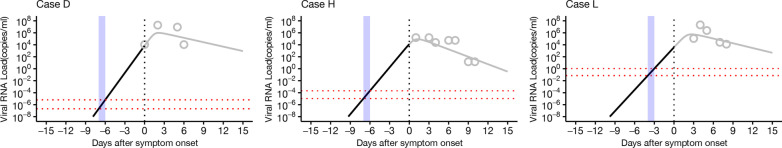
Viral load dynamics of the three patients in China. The three panel presents timeline of infection for the three cases in Zhuhai, China used to compute the viral load boundary for infection establishment. Expected SARS-CoV-2 viral dynamics and observed viral load are depicted by grey (or black) solid lines and grey open circles, respectively. The timing of symptom onset (black dashed lines), the timing of infection establishment (known; blue shaded areas), and the estimated viral load boundaries for infection establishment (red dashed lines) are also shown

To define “viral load boundary for infection establishment”, we used the information of the three secondary cases with known primary cases in Zhuhai (i.e., Patients D, H and L) [43]: a primary infected patient (Patient E) worked in Wuhan and visited Patient D and Patient L on January 17th, then Patients D and L developed symptoms on January 23rd and 20th, respectively. Other primary infected patients (Patients I and P) visited Patient H on January 11th, and fever developed in Patient H on January 17th. This implies that exposure started on the day when the primary cases visited those secondary cases. Assuming that infection established on the initial day of exposure in the secondary cases, we hindcasted the mathematical model, and obtained the viral load on the initial day of exposure, which is defined as the infection establishment boundary: 10^–6.67^ to 10^–5.18^, 10^–5.20^ to 10^–3.88^ and 10^–1.14^ to 10^0.03^ for Patients D, H and L, respectively. We used the lowest (10^–6.67^) and highest (10^0.03^) values as the boundary (Fig. 2).

### Nonlinear mixed-effects model

The nonlinear mixed-effects modelling approach incorporates fixed effects as well as random effects which describe the inter-patient variability in parameters. Including random effects amounts to a partial pooling of the data of all patients to improve estimates of the fixed effect parameters applicable across the cases. The parameter of patient $k$, ${\theta }_{k} (=\theta \times {e}^{{\pi }_{k}})$ is a product of $\theta$ (the fixed effect) and ${\pi }_{k}$ (the random effect), where ${\pi }_{k}$ is assumed to follow a normal distribution: $N(0,\Omega )$*.* The fixed effect parameters and random effect parameters were estimated using the stochastic approximation Expectation/Maximization (SAEM) algorithm and empirical Bayes method, respectively. A right-truncated normal distribution was used in the likelihood function to account for the left censoring of the viral load data (i.e., when the viral load is not detectable) [48]. MONOLIX 2019R2 (www.lixoft.com), a program for maximum likelihood estimation for a nonlinear mixed-effects model, was employed to fit the model to the viral load data. We changed the initial values multiple times to avoid local minimum of AIC and confirmed the robustness of parameter estimation.

## Results

Before describing the viral load data, here is the epidemiological situation when the data were collected. Figure 1A depicts the weekly epidemic curve in Singapore from January 21st to March 15th based on the onset of symptoms and laboratory confirmation. The epidemic curve based on laboratory confirmation follows the curve based on the onset of symptoms because of the reporting delay. For the first few weeks, the epidemic in Singapore was not in the phase of exponential growth, which suggests secondary transmissions were limited and any successive transmission did not take place yet. The first 18 cases discussed here are observed in the first two weeks of the epidemic.

For the first 12 ‘imported’ cases with travel history to Wuhan (6 were removed from the analysis due to insufficient viral load data or antiviral treatment. See the “Viral load data” section), their viral load data are plotted along with the curve of viral load estimated from the model (Fig. 1B) (note that time since the onset of symptoms was used as a time scale). Typically, the viral load exponentially increases since infection because the viruses reproduce themselves successively in the target cells. The viral load hits peak when uninfected target cells run out. Then the viral load starts to decrease over time as the viruses and infected cells are removed from the host body. Although the viral dynamics curves of different patients share the above characteristics, there was also huge heterogeneity in the dynamics. Especially, the virus persistence (i.e., the length of time the viral load is above the detection limit) and the hight of peak viral load were different between the patients. Such variability is due to the difference in biological (immunological) characteristics of each patient. For example, lower death rate of infected cells, $\delta$, is translated into longer virus persistence (slower decay in viral load) (Additional file 1: Table S1). However, interestingly, the viral load consistently hit the peak about 2–3 days after the onset of symptoms, suggesting viral shedding is high even before the clinical onset. Although viral shedding does not necessarily reflect the magnitude of infectiousness, this finding is concordance with the previous studies suggesting non-negligible amount of pre-symptomatic infection [12–16]. Notably He and colleagues demonstrated that the infectiousness profile peaks around the onset of symptoms [16], which is similar to the viral load dynamics we estimated in this study.

Hindcasting the estimated viral load dynamics, the day of infection establishment was estimated, where viral load reaches the boundary (indicated by shaded blue area in Fig. 1B). Note that the estimation of the day of infection establishment has some uncertainty (about 6 days) because of uncertainty on the boundary of viral load threshold. By comparing the day of infection establishment and the reported day of arrival in Singapore (which should be available from the immigration record in general), suggested by red dotted line in Fig. 1B, whether the case was infected in or out of Singapore was assessed. Additional file 1: Table S2 summarized the day of infection establishment and arrival to Singapore in calendar time. We found that 6 of the 12 cases (Case 2, 3, 4, 14, 16, 17) were clearly concluded as imported cases, whereas the remainder 6 cases (Case 6, 8, 9, 11, 12, 18) could have resulted from ongoing transmission locally in Singapore. For those suspicious secondary cases, contact tracing could provide further confirmation as to the timing of infection.

In fact, although all of the 12 were considered to be imported cases due to their travel history, the detailed investigation [42] revealed 3 cases (Case 8, 11, 18) among the 6 cases (Case 6, 8, 9, 11, 12, 18) for whom we could not exclude the possibility of secondary transmission had close contact with previously confirmed cases: Case 8 is a spouse of Case 9 travelled together to Singapore; Case 11 and 18 were identified as close contacts of Case 4 and 12, respectively. These findings suggest that our approach complements the traditional interview-based approach, and together with it, we can differentiate imported cases and cases of local transmission.

## Discussion

Many countries in Europe, Asia, and North America experienced several waves of COVID-19 epidemic, which were slowed down due to stringent lockdown measurements. The lockdown is not a sustainable measure because it downregulated world’s economy and impacted citizens’ health both physically and mentally [49, 50]. Therefore, ways to control epidemics without the lockdown measures have been warranted.

In order to avoid further waves of the epidemic without stringent lockdown measures, the following three measures should be implemented together: border control; test, trace contacts and isolate; and social distancing. Along with them, closely monitoring transmission mode by contact trace is essential to identify cases and avoid further transmission. However, the contact tracing is generally interview-based and the timing of infection inferred by this method is influenced by recall bias. We proposed viral load-based approach to help contact trace identify the timing of infection establishment.

Indeed, contact trace is widely conducted in many countries, however, the process is time-consuming. Using digital technology has been proposed to identify contacts faster [51], although there are issues of privacy and data protection [52–54]. The Singapore government released such application as a step toward lifting lockdown [55], however, only limited population have installed the application so far [56]. If many people do not install the app, all contacts cannot be identified. Further, infection events can occur indirectly (transmission can occur from contaminated surfaces [57, 58]), whether such apps can identify when and where infection occurred and eventually prevent outbreak is still uncertain.

In this study, we assessed whether the 12 initial cases which were classified as ‘imported’ were in fact imported or the result of secondary local transmission within Singapore. We found that 50% of the cases (6 out of 12) were clearly infected before arrival to Singapore (i.e., imported cases), the remaining half of the cases, however, could have been infected after the arrival in Singapore (i.e., secondary local transmission). This implies the possibility of within-country transmission prior to the 19th case (who is the result of local transmission) being reported on February 3rd. Combined with interview-based contact tracing, this approach can identify the trace of local secondary transmission.

Our method is useful to infer the timing of infection, discerning between cases imported or autochthonous (i.e., before or after arrival to the country) complementing contact trace. The advantage of using this method is that computation is solely based on viral load data. Collecting viral load could be a part of clinical practice as viral load has been collected for clinical purposes such as to understand the pathophysiology and aetiology of new diseases especially in the early phase of outbreak [59–62]. Further, viral load is measurable with patients’ saliva (not nasopharyngeal swabs, which has been widely used) in COVID-19 cases. It will reduce the effort of measuring viral load and the risk of infection for health practitioners [63, 64]. Given that recall bias is an issue of contact tracing, our method can assist inferring the timing of infection which is usually done by interview. In other words, estimation using our approach will be further enhanced if combined with the complementary information (e.g., travel and contact history and genetic information) thus reducing uncertainty in our predictions. Therefore, we empathize that we are not undermining the value of contact tracing.

There are a few limitations in our approach. First, our approach requires viral load data over multiple time points (only the patients with more than three viral load data points were included in the analysis); therefore, we may not be able to accurately estimate the timing of infection if the number of data points are limited. In addition to the number of data points, the timing of data collection (i.e., immediately after or long after symptom onset) will be the key that determines whether the dynamics can be accurately estimated. Future studies should argue data collection process to define the best practices for the use of the viral dynamics model in the context of inferring the timing of infection. Second, we need to note that both the boundaries and the day of infection establishment estimated using our approach could be underestimated, because infection is established after exposure starts. Third, we estimated the viral load boundaries of infection establishment with limited available data. Further studies need to investigate what factors are associated with the boundaries, such as countries, strains, and viruses. Fourth, we did not include the cases received antiviral therapy because of lack of information about its effect for SARS-CoV-2. However, once enough data about those antivirals are available, we can include those cases (we have already proposed a model accounting for antiviral effect [38, 65, 66]).

## Conclusions

Now is the time to think about the balance between risk of further waves and the social and economic damage accompanied by lockdown. To secure social and economic activities whilst controlling the risk of next wave, we need to closely monitor infection events as well as implement various preventive measurements, which were effective to hammer the waves of the epidemic. Contact trace plays an essential role. Collecting and analysing viral load data would help calibrate the timing of infection establishment estimated by contact tracing. As such, the method we used may be critical to help shape a country’s early response to the next wave.

## Supplementary Information

**Additional file 1: Table S1.** Estimated parameters for each case. **Table S2.** Date of arrival, symptom onset, estimated day of infection for Singapore cases infected with SARS-CoV-2.

## Data Availability

The data are publicly available from following two publications: Young BE, Ong SWX, Kalimuddin S, Low JG, Tan SY, Loh J, Ng OT, Marimuthu K, Ang LW, Mak TM et al.: Epidemiologic Features and Clinical Course of Patients Infected With SARS-CoV-2 in Singapore. JAMA 2020, 323(15):1488–1494. Zou L, Ruan F, Huang M, Liang L, Huang H, Hong Z, Yu J, Kang M, Song Y, Xia J et al.: SARS-CoV-2 Viral Load in Upper Respiratory Specimens of Infected Patients. N Engl J Med 2020, 382(12):1177–1179.
