# Supplementary material for: Estimation of timing of infection from longitudinal SARS-CoV-2 viral load data: mathematical modelling study
Source: BMC Infect Dis. 2022 Jul 28;22:656. doi: 10.1186/s12879-022-07646-2 (PMC9331019; doi:10.1186/s12879-022-07646-2)
Supplement: Supplementary file 1 — Additional file 1: Table S1. Estimated parameters for each case. Table S2. Date of arrival, symptom onset, estimated day of infection for Singapore cases infected with SARS-CoV-2. [file 12879_2022_7646_MOESM1_ESM.docx]

**Additional file 1**

Estimation of timing of infection from longitudinal SARS-CoV-2 viral load data: mathematical modelling study

Keisuke Ejima, Kwang Su Kim, Ana I. Bento, Shoya Iwanami, Yasuhisa Fujita, Kazuyuki Aihara, Kenji Shibuya, and Shingo Iwami

**Table S1. Estimated parameters for each case**

| **Zhuhai cases** |  |  |  |  |  |
| --- | --- | --- | --- | --- | --- |
| Case ID | $\gamma$ (day^-1^) | $\beta$ $(($copies/ml)^-1^ day^-1^) | $\delta$ (day^-1^) | $V\left( 0 \right)$ (copies/ml) | $T_{inf}$ (day)^*^ |
| C | 3.09 | 1.90×10^-5^ | 0.80 | 7.34×10^3^ | -9.8, -3.6 |
| D | 4.01 | 0.24×10^-5^ | 0.57 | 7.00×10^3^ | -7.0, -2.5 |
| E | 3.08 | 1.41×10^-5^ | 0.66 | 6.52×10^3^ | -9.5, -3.5 |
| H | 3.87 | 1.77×10^-5^ | 1.07 | 1.07×10^4^ | -8.1, -3.1 |
| I | 3.76 | 0.82×10^-6^ | 0.42 | 3.65×10^3^ | -7.0, -2.4 |
| L | 3.33 | 0.30×10^-5^ | 0.63 | 3.77×10^3^ | -8.7, -3.0 |
| N | 3.14 | 1.05×10^-5^ | 0.58 | 5.72×10^3^ | -9.1, -3.3 |
| O | 2.91 | 5.22×10^-5^ | 1.46 | 3.47×10^4^ | -6.3, -2.7 |
| P | 3.76 | 0.70×10^-5^ | 0.95 | 5.66×10^4^ | -8.4, -3.0 |
| Q | 3.12 | 1.11×10^-5^ | 0.60 | 5.81×10^4^ | -9.3, -3.3 |
| S | 3.08 | 1.19×10^-5^ | 0.51 | 6.20×10^4^ | -9.1, -3.3 |
| T | 3.03 | 1.95×10^-5^ | 0.90 | 5.59×10^4^ | -10.6, -3.8 |
| Median | 3.13 | 1.15×10^-5^ | 0.64 | 6.01×10^4^ | -8.9, -3.2 |
| **Singapore cases** |  |  |  |  |  |
| Case ID | $\gamma$ (day^-1^) | $\beta$ $(($copies/ml)^-1^ day^-1^) | $\delta$ (day^-1^) | $V\left( 0 \right)$ (copies/ml) | $T_{inf}$ (day)^*^ |
| 2 | 2.78 | 1.44×10^-5^ | 0.62 | 3.99×10^3^ | -10.6, -3.7 |
| 3 | 3.64 | 0.15×10^-5^ | 0.42 | 4.01×10^3^ | -7.4, -2.6 |
| 4 | 3.11 | 0.97×10^-5^ | 0.63 | 5.24×10^3^ | -9.4, -3.4 |
| 6 | 3.53 | 0.49×10^-5^ | 0.41 | 5.25×10^3^ | -7.6, -2.7 |
| 8 | 2.11 | 2.32×10^-5^ | 0.33 | 3.08×10^3^ | -12.6, -4.3 |
| 9 | 2.53 | 2.99×10^-5^ | 0.22 | 6.30×10^3^ | -9.6, -3.5 |
| 11 | 3.79 | 1.52×10^-5^ | 1.02 | 1.01×10^4^ | -8.3, -3.1 |
| 12 | 3.08 | 1.43×10^-5^ | 0.68 | 6.37×10^3^ | -9.6, -3.5 |
| 14 | 3.41 | 0.94×10^-6^ | 0.89 | 2.05×10^3^ | -9.1, -3.0 |
| 16 | 3.20 | 0.74×10^-5^ | 0.48 | 5.27×10^3^ | -8.7, -3.1 |
| 17 | 2.32 | 3.56×10^-5^ | 0.74 | 2.09×10^3^ | -13.6, -4.5 |
| 18 | 3.20 | 0.82×10^-5^ | 0.35 | 4.79×10^3^ | -8.3, -2.9 |
| Median | 3.15 | 1.20×10^-5^ | 0.55 | 5.01×10^3^ | -9.3, -3.3 |

^*^ Maximum and minimum days before symptom onset

**Table S2. Date of arrival, symptom onset, estimated day of infection for Singapore cases infected with SARS-CoV-2**

| Case ID | Date of arrival to Singapore | Date of symptom onset | Length of infection to arrival (days) | Estimated day of infection establishment |
| --- | --- | --- | --- | --- |
| 2 | 1/20/2020 | 1/21/2020 | 3.5 to 12.6 | 1/07/2020 to 1/16/2020 |
| 3 | 1/20/2020 | 1/14/2020 | 9.7 to 16.6 | 1/03/2020 to 1/10/2020 |
| 4 | 1/22/2020 | 1/22/2020 | 2.6 to 7.4 | 1/14/2020 to 1/19/2020 |
| 6 | 1/19/2020 | 1/25/2020 | -2.6 to 3.4 | 1/15/2020 to 1/21/2020 |
| 8 | 1/19/2020 | 1/25/2020 | -3.3 to 1.6 | 1/17/2020 to 1/22/2020 |
| 9 | 1/19/2020 | 1/24/2020 | -0.7 to 7.6 | 1/11/2020 to 1/19/2020 |
| 11 | 1/22/2020 | 1/27/2020 | -1.5 to 4.6 | 1/17/2020 to 1/23/2020 |
| 12 | 1/22/2020 | 1/26/2020 | -0.9 to 4.3 | 1/17/2020 to 1/22/2020 |
| 14 | 1/26/2020 | 1/29/2020 | 0.5 to 6.6 | 1/19/2020 to 1/25/2020 |
| 16 | 1/22/2020 | 1/23/2020 | 2.0 to 8.1 | 1/13/2020 to 1/20/2020 |
| 17 | 1/30/2020 | 1/30/2020 | 3.1 to 8.7 | 1/21/2020 to 1/26/2020 |
| 18 | 1/23/2020 | 1/30/2020 | -4.1 to 1.3 | 1/21/2020 to 1/27/2020 |
